# Supplementary material for: A Humanized Yeast Phenomic Model of Deoxycytidine Kinase to Predict Genetic Buffering of Nucleoside Analog Cytotoxicity
Source: Genes (Basel). 2019 Sep 30;10(10):770. doi: 10.3390/genes10100770 (PMC6826991; doi:10.3390/genes10100770)
Supplement: Supplementary file 1 [file genes-10-00770-s001.zip › Additional_Files/Additional_File7_GOTermHeatmaps.zip!/Additional_File7_GOTermHeatmaps/mitotic_G2_M_transition_checkpoint.pdf]

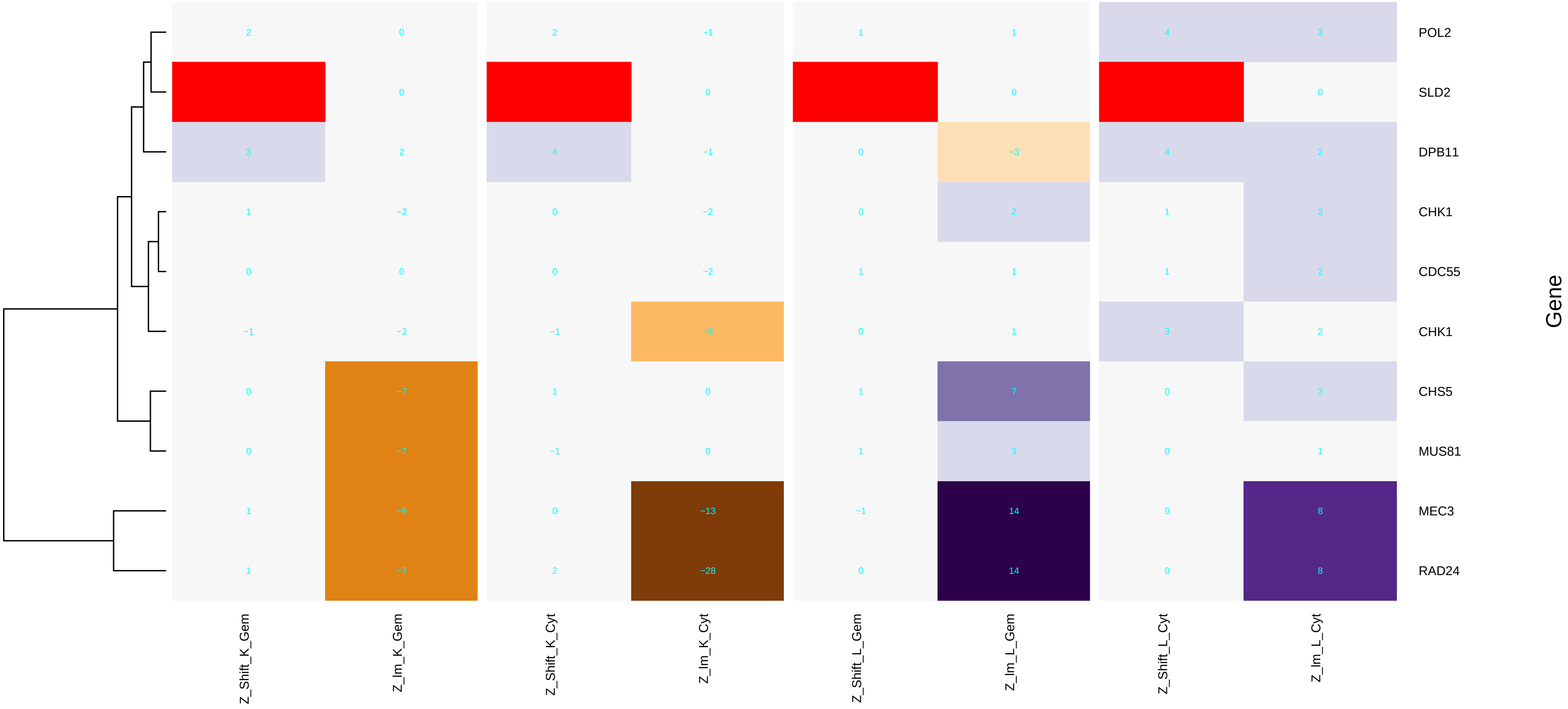
POL2: 2 0 2 −1 1 1 4 3
SLD2: 0 0 0 0
DPB11: 3 2 4 −1 0 −3 4 2
CHK1: 1 −2 0 −2 0 2 1 3
CDC55: 0 0 0 −2 1 1 1 2
CHK1: −1 −2 −1 −6 0 1 3 2
CHS5: 0 −7 1 0 1 7 0 3
MUS81: 0 −7 −1 0 1 3 0 1
MEC3: 1 −6 0 −13 −1 14 0 8
RAD24: 1 −7 2 −28 0 14 0 8
Z_Shift_K_Gem
Z_Im_K_Gem
Z_Shift_K_Cyt
Z_Im_K_Cyt
Z_Shift_L_Gem
Z_Im_L_Gem
Z_Shift_L_Cyt
Z_Im_L_Cyt
Gene
